# Two Novel Arenaviruses Detected in Pygmy Mice, Ghana

**DOI:** 10.3201/eid1911.121491

**Published:** 2013-11

**Authors:** Karl C. Kronmann, Shirley Nimo-Paintsil, Fady Guirguis, Lisha C. Kronmann, Kofi Bonney, Kwasi Obiri-Danso, William Ampofo, Elisabeth Fichet-Calvet

**Affiliations:** US Naval Medical Research Unit No. 3, Ghana Detachment, Accra, Ghana (K.C. Kronmann, S. Nimo-Paintsil, L.C. Kronmann);; US Naval Medical Research Unit No. 3, Cairo, Egypt (F. Guirguis);; Noguchi Memorial Institute for Medical Research, Legon, Ghana (K. Bonney, W. Ampofo, S. Nimo Paintsil);; Kwame Nkrumah University of Science and Technology, Kumasi, Ghana (K. Obiri-Danso);; Bernhard Nocht Institute of Tropical Medicine, Hamburg, Germany (E. Fichet-Calvet);; Evolutionary Biology Group, University of Antwerp, Antwerp, Belgium (E. Fichet-Calvet)

**Keywords:** arenaviruses, viruses, Lassa virus, Ghana, Murinae, pygmy mice, Rodentia, zoonoses, reservoirs, emerging communicable diseases, epidemiology, Lassa fever, Western Africa, west Africa

## Abstract

Two arenaviruses were detected in pygmy mice (*Mus* spp.) by screening 764 small mammals in Ghana. The Natal multimammate mouse (*Mastomys natalensis*), the known Lassa virus reservoir, was the dominant indoor rodent species in 4 of 10 sites, and accounted for 27% of all captured rodents. No rodent captured indoors tested positive for an arenavirus.

Lassa fever is an arenavirus infection transmitted to humans from rodents in a limited geographic region of western Africa. Nosocomial outbreaks have been recorded in Sierra Leone, Liberia, Guinea, and Nigeria: the countries best known to report Lassa fever (*1*). Most cases reported in travelers have originated in these 4 countries (*2*). However, cases have been reported from other countries in the region, including 1 caused by a previously undescribed strain of Lassa virus (LASV) after the case-patient traveled through Ghana (*3*). In addition, infection of humans with an arenavirus other than LASV has recently been recognized in southern Africa (*4*).

LASV has a bisegmented genome: the nucleoprotein (NP) and glycoprotein (GP) genes are on the small RNA segment, and the polymerase (L) and matrix protein (Z) genes are on the large RNA segment. LASV circulates in rodent populations even when infections in humans are not occurring, providing a source for subsequent outbreaks among humans.

Arenaviruses have species-specific reservoirs, and studies in Sierra Leone and Guinea found *Mastomys natalensis* to be the only rodent reservoir for LASV (*5*,*6*). In Guinea, *M. natalensis* abundance and viral prevalence rates in rodents have been associated with LASV seroprevalence among humans (*6*). Using a risk map model, we selected 10 sites in Ghana to examine rodent populations and arenavirus carriage rates. 

## The Study

The study was performed in accordance with a protocol approved by the Institutional Review Board of the Noguchi Memorial Institute for Medical Research, and the Institutional Review Board and Institutional Animal Care and Use Committee of the US Naval Medical Research Unit No. 3.

**Figure 1:**
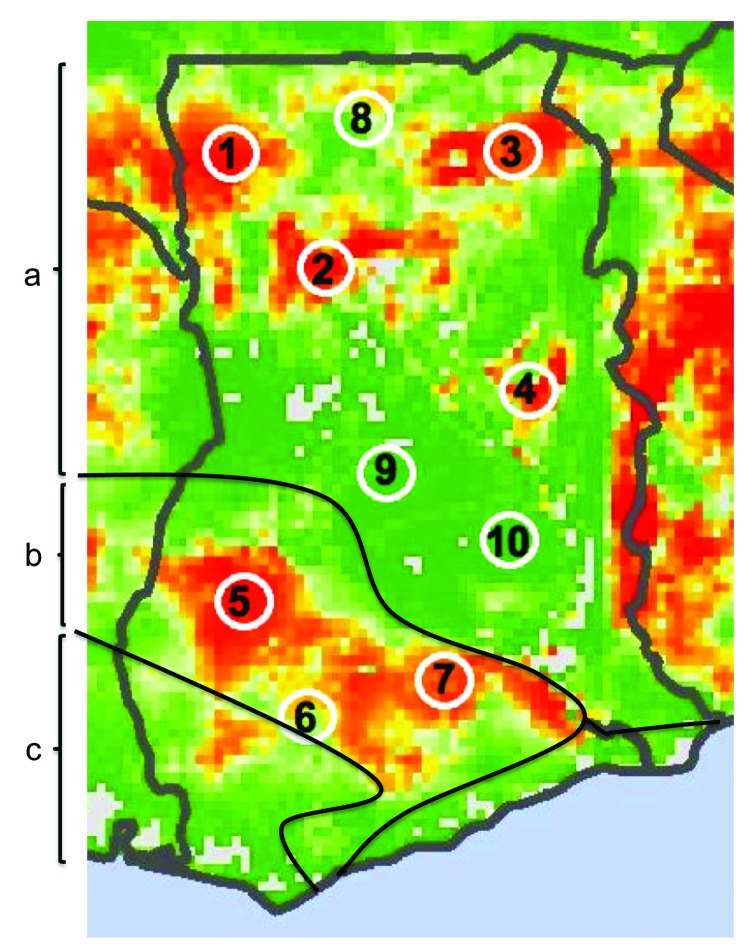
Lassa virus risk map of Ghana showing 10 numbered study sites adapted from Fichet-Calvet and Rogers, Model 3 (*1*). Red areas indicate high predicted risk for Lassa fever and green areas indicate low predicted risk. Solid black lines and letters indicate vegetation zones: a) Guinea savanna woodland; b) moist semideciduous forest; c) tropical rainforest.

Seven sites were selected from areas of high predicted risk and 3 from areas of low predicted risk (Figure 1). A village was then selected for each site according to 3 criteria: a human population between 500 and 2,000; distance >20 km from any urban center or major road; and willingness to participate. All field work was scheduled during the rainy seasons of 2010 and 2011, when viral prevalence rates in rodents have been shown to be higher (*7*).

Traps were baited and set overnight in houses and outside in fields and woods along trap lines. Rodents captured were necropsied on site by using field biosafety level 3 procedures (*8*). Morphologic data, blood, and organs were collected from each animal for species identification and viral testing.

A total of 16 species of rodents and 1 genus of shrews were identified among 764 captures (Table). *Mastomys natalensis*, the target species for the study, represented 27% (209/764) of captured species, and was outnumbered by another commensal rodent, *Praomys daltoni*, in 6 of 10 sites (Table). Trapping success was calculated for traplines set on 3 consecutive nights (no. trapped rodents ÷ no. of traps set per night × no. of nights) and was 9.2% (635/6895) for all locations; 23% indoors and 4.8% outdoors. Of rodents captured indoors, 98% (492/504) were *Mastomys* or *Praomys* species.

**Table:** Species distribution of small mammals captured by village in Ghana*

Species	No. animals captured, by study site (indoors/total)

1	2	3	4	5	6	7	8	9	10	All sites
*Crocidura* spp.	1/1	0	0/1	0/1	0	0/3	1/7	1/5	2/3	0	5/21
* Gerbilliscus gambianus*	0/1	0	0	0	0	0	0	0	0	0	0/1
* Gerbilliscus kempi*	0	0/1	0	0/4	0	0	0	0	0	0	0/5
* Lemniscomys striatus*	0	0	0	0	0	0	0/2	0	0	0	0/2
* Lophuromys sikapusi*	0	0	0	0	0	0	0/3	0	0	0	0/3
* Mastomys erythroleucus*	0/3	0	0	0	0	0/1	0/6	0	0/2	0/10	0/22
* Mastomys natalensis*	30/33	54/59	1/1	11/14	4/10	0	0/3	32/32	35/40	13/17	180/209
* Mus baoulei*	0	0	0	0/2†	0	0	0	0	0	0/3	0/5
* Mus mattheyi*	1/12†	0/4	0/7	0/21	0	0	0	1/8	0/13	0/24	2/89
* Mus minutoides*	0	0	0	0	0	0/16	0/7	0	0/5	0/1	0/29
* Mus musculoides*	1/1	0	0	0	0	0	0	0/4	0	0	1/5
* Mus setulosus*	0	0	0	0	0/1	0	0/4	0	0	0	0/5
* Praomys daltoni*	0/2	1/5	55/57	51/53	56/57	60/60	55/57	0/4	5/5	29/31	312/331
* Praomys tullbergi*	0	0	0	0	0	0	0/2	0	0	0	0/2
* Rattus rattus*	0	0	0	0	0	1/1	2/3	0	1/1	0	4/5
* Taterillus gracillis*	0/12	0	0/2	0/5	0	0	0	0/1	0/9	0	0/29
* Uranomys ruddi*	0	0	0	0	0	0	0	0	0	0/1	0/1
Total	33/65	55/69	56/68	62/100	60/68	61/81	58/94	34/54	43/78	42/87	504/764

*Site names and coordinates: 1 = Natorduori (10°15.49′N 02°37.56′W), 2 = Bowena (09°32.82′N 01°37.95′W), 3 = Teanoba (10°23.70′N 00°21.96′W), 4 = Jirandogo (08°20.83′N 00°20.75′W), 5 = Amomaso (07°07.36′N 02°19.66′W), 6 = Ankaakur (06°10.56′N 01°47.56′W), 7 = Ehiawenwu (06°26.97′ N 00°51.11′ W), 8 = Doninga (10°37.16′N 01°25.31′W), 9 = Mangoase (07°58.11′N 01°39.79′W), 10 = Monkwo (07°40.45′N 00°37.98′W). † rodent with positive PCR for arenavirus.

Total RNA was extracted from whole blood, or homogenized heart tissue when blood was not sufficient, in the biosafety level 3 facility at Noguchi Memorial Institute for Medical Research by using the RNeasy Mini Kit (QIAGEN, Hilden, Germany) with QIAshredder columns and purified with on-column RNase-free DNase set (QIAGEN). The samples were tested for the presence of arenavirus GP gene RNA by using Power SYBR Green RNA-to-C 1-Step PCR Kit (Applied Biosystems, Foster City, CA, USA) (*9*).

To confirm the first screening, a second PCR targeting the L gene was performed in the *Mastomys* and *Mus* spp. samples (*10*). Two *Mus* spp, caught outdoors in the villages of Jirandogo (site 4) and Natorduori (site 1), respectively, were found to be positive for arenavirus RNA. For these 2 positive specimens, additional PCRs were performed by using primers OWS1+, OWS1000-, OWS2165A+, OWS2165B+, OWS2840A-, OWS2840B-, OWS2770+, OWS3400A-, and OWS3400- to acquire longer fragments of GP and NP genes (*11*). These fragments were sequenced on both strands, assembled, and aligned in MacVector (MacVector, Inc., Cary, NC, USA), then phylogenetically analyzed using PhyML (*12*). The viral and murine (cytochrome b) sequences were deposited in GenBank under accession nos. JX845167–JX845174. Voucher specimens are stored at Noguchi Memorial Institute for Medical Research and the United States Army Medical Research Institute for Infectious Diseases.

**Figure 2:**
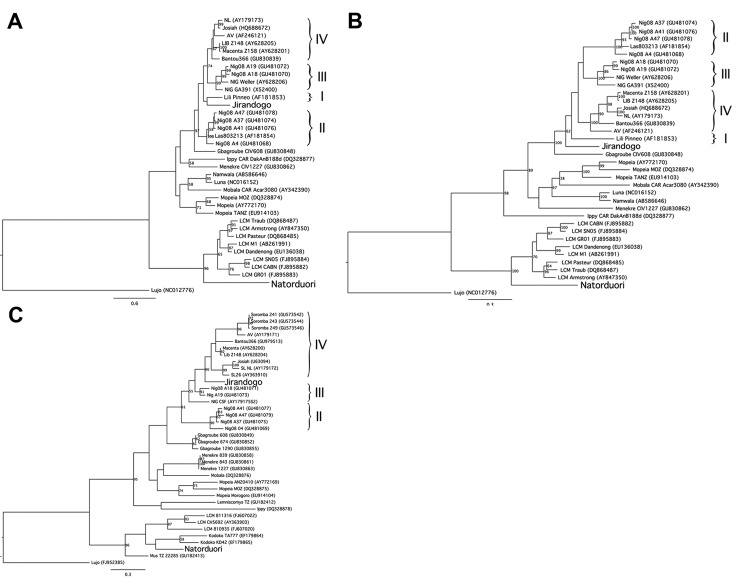
Phylogenetic trees depicting virus sequences found in rodents from the villages of Jirandogo and Natorduori, Ghana. Lineages of Lassa virus clade are indicated by Roman numerals on the right. For each virus, phylogenetic trees are shown for 3 genes: 2a, glycoprotein gene (partial 1,034 bp), 2b, nucleoprotein gene (partial 1,297 bp), and 2c, Polymerase gene (L partial, 340 bp). The analysis was performed using PhyML (*11*), with a general time reversible nucleotide substitution model and 100 bootstrap replicates. Branches highly supported by PhyML are indicated with bootstrap values >50. Scale bars indicate nucleotide substitutions per site.

The phylogenetic position of the virus found in a *Mus baoulei* mouse, named Jirandogo for the village in which it was discovered, is unclear: Jirandogo clusters with the Nigerian LASV strain Lili Pinneo (lineage I) but with low branch support (20% bootstrap support) in the GP gene tree (Figure 2, panel A); is basal to all Lassa strains in the NP gene. At the nucleotide level, identity scores between Jirandogo and other published Lassa strains were 70.9%–74.6%, 71.6%–74.6%, and 76.9%–83.8% for GP, NP, and L, respectively. At the amino acid level, the scores ranged between 79.1%–84.2%, 82.0%–84.2%, and 93.8%–98.2% for GP, NP and L proteins, respectively. The other sequence, found in *Mus mattheyi* mice and named Natorduori after the village in which it was found, clusters with lymphocytic choriomeningitis virus in all 3 phylogenetic trees.

## Conclusions

This study aimed to provide data on the risk for Lassa fever in Ghana, a country situated between well-known Lassa fever–endemic regions, but with little known disease itself. Our findings support the idea that Lassa virus is not widely prevalent in Ghana, and the *Mastomys natalensis* reservoir is not as common as in more highly endemic countries. In Ghana, 27% (209 of 764 captured rodents) were identified as *Mastomys natalensis*, compared with 54% (601/1123) in Guinea and 80% (82/103) in Mali (*7*,*13*). Overall, *P. daltoni*, a species not known to harbor LASV, outnumbered *M. natalensis* in Ghana, and was the predominant rodent species found indoors in 6 of our 10 sites.

Sequences of arenaviruses were detected in 2 species of pygmy mice, *Mus baoulei* and *Mus mattheyi*. Jirandogo, the sequence found in *Mus baoulei*, is phylogenetically close to LASV clade viruses. However, the maximum amino acid difference of 18% in NP between Jirandogo and LASV exceeds the 12% cutoff criteria, and therefore places it outside the LASV clade (*14*).

Arenaviruses other than LASV have recently been reported to cause human disease in Africa, but it is not known whether the viral sequences we found in Ghana are from viruses pathogenic to humans. *Mus* species in Africa are sometimes found indoors where exposure to humans is more likely, but most are found outdoors, including those from which we collected the 2 positive samples tested in our study. Infrequent cases in humans would be expected from arenavirus carriage in outdoor species, such as *Mus*.

It is notable that possible cases of Lassa fever have recently been reported in Ghana by the Ministry of Health from the high risk area near site 5 of our study (*4*). These reported cases in humans occurred ≈35 km from site 5, where *Mastomys natalensis* represented 15% of all rodents captured, and showed no evidence of arenavirus infection by PCR testing. The cases in humans were reported on the basis of the results of PCR tests, and further sequencing will be necessary to confirm the finding.

Although arenaviruses have species–specific reservoirs, recent work suggests host switching may be more common than previously believed (*15*). It is reassuring that no arenavirus was found in *Mastomys natalensis* species tested from Ghana, although 2 were detected in other rodent species from sites with high predicted risk. More information about geographic and temporal fluctuations in *Mastomys natalensis* rodent populations, the frequency of virus host-switching among rodents and the degree of arenavirus circulation is needed to better understand the implications of our findings for the risk for disease outbreaks from LASV or other arenaviruses in Ghana.
